# Timeliness of Surveillance during Outbreak of Shiga Toxin–producing *Escherichia coli* Infection, Germany, 2011

**DOI:** 10.3201/eid1710.111027

**Published:** 2011-10

**Authors:** Mathias Altmann, Maria Wadl, Doris Altmann, Justus Benzler, Tim Eckmanns, Gérard Krause, Anke Spode, Matthias an der Heiden

**Affiliations:** Author affiliations: Robert Koch Institute, Berlin, Germany (M. Altmann, M. Wadl, D. Altmann, J. Benzler, T. Eckmanns, G. Krause, M. an der Heiden);; Health Department of the Hamburg Northern District, Hamburg, Germany (A. Spode)

**Keywords:** bacteria, foodborne infections, Shiga toxin–producing Escherichia coli, Escherichia coli, surveillance, timeliness, notification, reporting, STEC, HUS, hemolytic uremic syndrome, enteric infections, Germany, dispatch, expedited

## Abstract

In the context of a large outbreak of Shiga toxin–producing *Escherichia coli* O104:H4 in Germany, we quantified the timeliness of the German surveillance system for hemolytic uremic syndrome and Shiga toxin–producing *E. coli* notifiable diseases during 2003–2011. Although reporting occurred faster than required by law, potential for improvement exists at all levels of the information chain.

In May and June 2011, Germany experienced the largest outbreak of hemolytic uremic syndrome (HUS) and bloody diarrhea related to Shiga toxin–producing *Escherichia coli* (STEC) ever reported (*1**,**2*). As of June 20, a total of 2,518 STEC cases and 786 HUS cases meeting the case definitions for this outbreak were reported to the national agency for infectious disease epidemiology (Robert Koch Institute [RKI]) through the surveillance system in Germany for notifiable diseases (GSSND) (*3*). The first outbreak-associated case-patient fell ill on May 1, followed by a sharp increase in the number of HUS case-patients on May 9 (by onset of symptom). Case numbers by disease onset peaked around May 22. Epidemiologic and food trace-back investigations identified fenugreek sprouts, grown from seeds probably contaminated by STEC, at a farm in Lower Saxony to be the vehicle of the outbreak (*4*). From June 10 on, German authorities recommended that raw sprouts should not be eaten.

In the GSSND, heads of laboratories have to send notification of STEC cases, and clinicians are legally mandated to report HUS cases within 24 hours to the local health department by fax, telephone, or letter (*5*). Legally, reporting of these cases from the local health department through the state health department to RKI must be completed within 16 days. To minimize the reporting delay, beginning May 23 the local health departments and state health departments agreed to report cases every working day (*6*).

On May 18, the first outbreak-associated case (patient’s onset of diarrhea was May 2) was reported to RKI. On the same day, a local hospital notified the local health department of Hamburg North about a cluster of HUS in 3 children. RKI was alerted to the outbreak cluster on May 19 by email.

Given the extent of the outbreak, questions arose about the timeliness of the GSSND regarding STEC and HUS cases. We assessed intervals between notifying and reporting STEC and HUS cases from January 1, 2003, through June 22, 2011, to identify potential needs and strategies for improvement.

## The Study

We divided the analysis into 3 periods: period A (before the outbreak) was from January 1, 2003, through April 30, 2011; period B (early phase of the outbreak) was from May 1 (when the first outbreak-associated case-patient fell ill) through May 18 (date when the HUS cluster was detected); and period C (late phase of the outbreak) was from May 19 through June 22. Data on timeline events for all reported STEC and HUS cases were collected from the GSSND (*7*). Timeline events comprised the following dates: symptom onset (onset of diarrhea), diagnosis, notification (date when the notification arrived at the local health department), and reporting (date when the report arrived at RKI). Dates of symptom onset and diagnosis were excluded when they were after the date of notification. For each case, intervals between timeline events were calculated from the dates available. Intervals were then assigned to 1 of the 3 periods (A, B, or C) according to the first date of the interval. Median times and interquartile ranges (IQR) were calculated in days for each type of interval for each period. Statistical analyses were done by using Stata software version 11.0 (StataCorp LP, College Station, TX, USA).

For the 1,394 HUS cases with available information, the median times from symptom onset in patients to diagnosis and to notification were similar in periods A and B (8 days and 9–10 days, respectively) and shorter in period C (4 and 5 days, respectively) (Table 1). The median time from symptom onset to reporting decreased from 20 days in period A to 12 and 8 days in periods B and C, respectively. The median time from diagnosis to notification was longer in period B (4.5 days) than in periods A and C (1 and 0 day, respectively). Among the 14 HUS cases with available information for period B, 10 (71%) were notified after 24 hours. The interval from notification of the local health department to report to RKI was longer in periods A and B than in period C (7 and 8 days vs. 3 days, respectively). For the 13,400 STEC cases with available information, we noticed in period B a longer delay from symptom onset in patients to reporting (15 days vs. 12 days for HUS) but a shorter delay from diagnosis to notification (2 days vs. 4.5 days for HUS) (Table 2). The Figure shows the increasing numbers of patients with disease onset on May 9, diagnosis and notification on May 18, and reporting on May 24.

**Table 1:** Median reporting intervals, in days, for 1,394 hemolytic uremic syndrome cases in 3 periods, Germany*

Interval†	Period A			Period B			Period C		Total no./N‡ (%)
	No./N‡ (%)	Med (IQR)		No./N‡ (%)	Med (IQR)		No./N‡ (%)	Med (IQR)	
From symptom onset to									1,267/1,394§ (91)
Diagnosis	283/497 (57)	8 (4–12)		136/237 (57)	8 (6–9)		331/533 (62)	4 (2–6)	750/1,267 (59)
Notification to LHD	312/497 (63)	10 (6–15)		173/237 (73)	9 (7–10)		396/533 (74)	5 (3–7)	881/1,267 (70)
Report to RKI	497/497 (100)	20 (14–26)		237/237 (100)	12 (9–15)		533/533 (100)	8 (6–11)	1,267/1,267 (100)
From diagnosis to									798/1,394§ (57)
Notification to LHD	264/294 (90)	1 (0–3)		14/15 (93)	4.5 (1–7)		473/489 (97)	0 (0–1)	751/798 (94)
Report to RKI	294/294 (100)	10 (7–16)		15/15 (100)	8 (8–9)		489/489 (100)	3 (2–6)	798/798 (100)
From notification to LDH to									943/1,394§ (68)
Report to RKI	319/319 (100)	7 (5–13)		6/6 (100)	8 (6–8)		618/618 (100)	3 (1–5)	943/943 (100)

*Period A, 2003 Jan 1–2011 Apr 30; Period B, 2011 May 1–2011 May 18; Period C, 2011 May 19–2011 Jun 22; med, median; IQR, interquartile range; LHD, local health department; RKI, Robert Koch Institute. †Classification of the interval in 1 of the 3 periods according to the first date of this interval. ‡No./N, no. patients having available data for both dates of the interval/no. patients having available data for the first date of the interval. §No. patients having available data for this date/total no. hemolytic uremic syndrome cases.

**Table 2:** Median reporting intervals, in days, for 13,400 Shiga toxin–producing *Escherichia coli* cases in 3 periods, Germany*

Interval†	Period A			Period B			Period C		Total no./N‡ (%)
	No./N‡ (%)	Med (IQR)		No./N‡ (%)	Med (IQR)		No./N‡ (%)	Med (IQR)	
From symptom onset to									9,365/13,400§ (70)
Diagnosis	4,734/6,700 (71)	8 (5–16)		368/494 (74)	9 (6–13)		1,606/2,171 (74)	4 (3–6)	6,708/9,365 (72)
Notification to LHD	4,652/6,700 (69)	11 (7–18)		423/494 (86)	10 (7–15)		1,848/2,171 (85)	5 (3–7)	6,923/9,365 (74)
Report to RKI	6,700/6,700 (100)	20 (14–30)		494/494 (100)	15 (11–20)		2,171/2,171 (100)	9 (6–12)	9,365/9,365 (100)
From diagnosis to									9,261/13,400§ (69)
Notification to LHD	6,088/6,802 (90)	1 (0–3)		69/70 (99)	2 (0–6)		2,353/2,389 (98)	0 (0–1)	8,510/9,261 (92)
Report to RKI	6,802/6,802 (100)	9 (6–14)		70/70 (100)	9.5 (7–13)		2,389/2,389 (100)	4 (2–6)	9,261/9,261 (100)
From notification to LDH to									9,529/13,400§ (71)
Report to RKI	6,712/6,712 (100)	7 (4–11)		50/50 (100)	8 (6–11)		2,767/2,767 (100)	3 (1–5)	9,529/9,529 (100)

*Period A, 2003 Jan 1–2011 Apr 30; Period B, 2011 May 1–2011 May 18; Period C, 2011 May 19–2011 Jun 22; med, median; IQR, interquartile range; LHD, local health department; RKI, Robert Koch Institute. †Classification of the interval in 1 of the 3 periods according to the first date of this interval. ‡No./N, no. patients having available data for both dates of the interval/no. patients having available data for the first date of the interval. §No. patients having available data for this date/total number of Shiga toxin–producing *Escherichia coli* cases.

**Figure:**
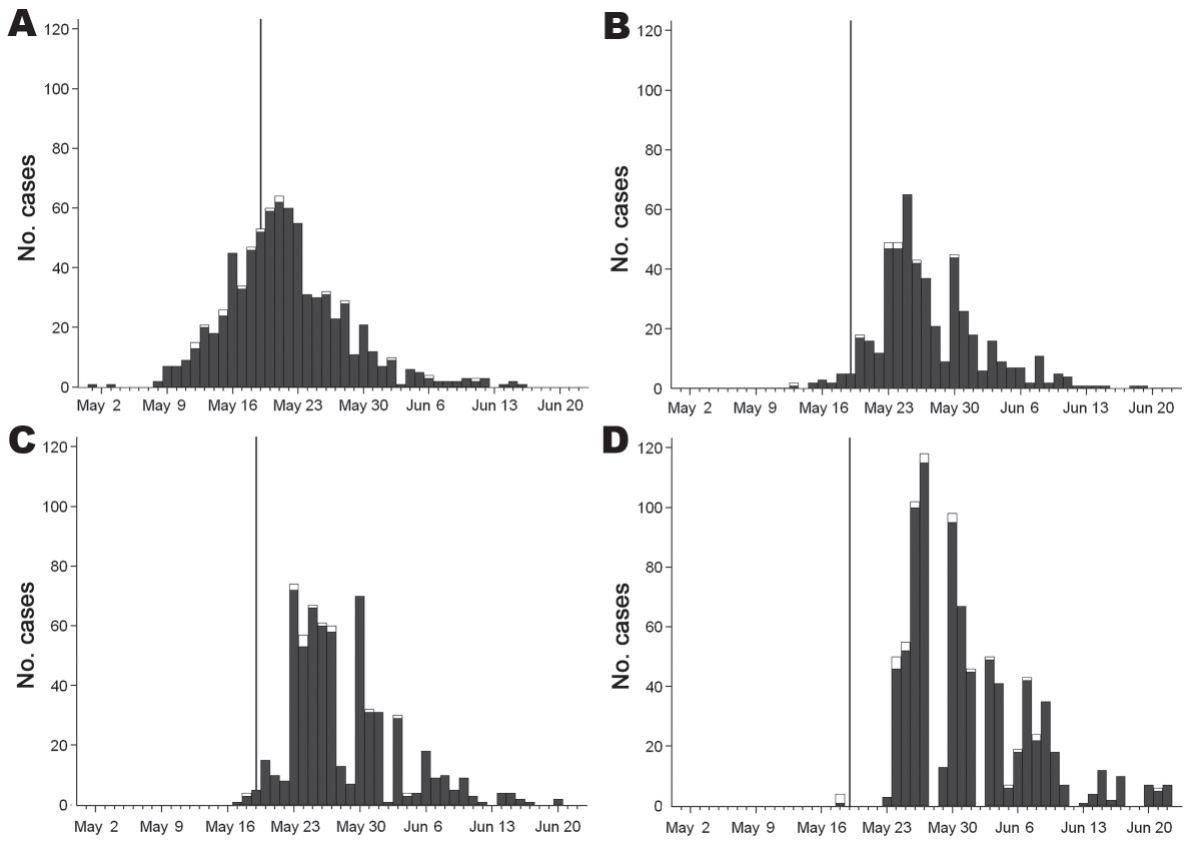
Hemolytic uremic syndrome (HUS) cases by date of symptom onset (A), date of diagnosis (B), date of notification (C) (i.e., the date that the local health department was notified of the case), and date of reporting (D) (i.e., the date that the Robert Koch Institute received the report of the case from the local health department) during outbreak of Shiga toxin–producing *Escherichia coli* infection and HUS, May–June 2011, Germany. Vertical lines indicate May 19, when the Robert Koch Institute received reports about a cluster of HUS cases in children. Dark gray bars represent outbreak-related cases; white bars represent cases not related to the outbreak. Only cases with available information are represented.

## Conclusions

A median of 11 days passed between onset of symptoms and notification of STEC cases in period A. A study by Hedberg et al. in 6 US states reported a delay of 7 days for the same interval for *E. coli* O157 infections (*8*). Considering that period B is biased for the interval “onset of symptom to reporting,” because the second date of the interval is likely to be in period C when the reporting flow was accelerated, we could only consider period A for this interval. We found that a median of 20 days occurred between symptom onset and reporting for STEC and HUS cases. This result is comparable to the 18 days reported for foodborne infections in the Netherlands (*9*). However, the duration between symptom onset and reporting can be reduced to 8 days, as was seen in period C. We also found that most of the HUS cases in period B were notified later than mandated by law. Although the number of cases was small, this is a remarkable result. It might be explained by the limited experience of nephrologists in notifying adult HUS cases. However, this also shows the need to motivate and to assist clinicians to notify within 24 hours (e.g., with an automatic electronic notification tool that could alert clinicians of their obligation to notify the disease when entering the diagnosis of HUS). By looking only at the timeline events directly under control of public health authorities, we found that the interval from notification of the local health department to reporting to RKI could be shortened from 1 week to 3 days if the local health department and the state health department routinely transmitted data on a daily basis.

This outbreak is a good example of circumstances in which single cases occur initially in multiple local health administrations in different federal states. In such situations, early outbreak detection and investigation become crucial to ensure early and continuous reporting to authorities at the national level. Given the current delays in diagnosis, notification, and reporting, this outbreak would have been detected at the national level considerably later than May 19 if the Hamburg health department had not promptly contacted RKI. This illustrates that state health departments and RKI need to receive local notifications earlier to successfully apply detection algorithms that would indicate potential multicounty or multistate outbreaks (*10*).

A revision of the notification and reporting system should be considered in Germany, with the goal of timely detection of increases in infectious diseases while being sustainable and specific. This result could be achieved if physicians and heads of laboratories could feed their data into a centralized database shared by local health departments, state health departments, and RKI with different access rights.
